# Organic Remobilization of zinc and phosphorus availability to plants by application of mineral solubilizing bacteria *Pseudomonas aeruginosa*

**DOI:** 10.1016/j.heliyon.2023.e22128

**Published:** 2023-11-11

**Authors:** K. Sunitha kumari, S.N. Padma Devi, Rajamani Ranjithkumar, Sinouvassane Djearamane, Lai-Hock Tey, Ling Shing Wong, Saminathan Kayarohanam, Natarajan Arumugam, Abdulrahman I. Almansour, Karthikeyan Perumal

**Affiliations:** a Department of Botany, PSGR Krishnammal College for Women, Peelamedu, Coimbatore-641 004, Tamil Nadu, India; b Viyen Biotech LLP, Coimbatore - 34, Tamil Nadu, India; c Biomedical Research Unit and Lab Animal Research Centre, Saveetha Dental College, Saveetha Institute of Medical and Technical Sciences, Saveetha University, Chennai 602 105, India; d Faculty of Science, Universiti Tunku Abdul Rahman, Jalan Universiti, Bandar Barat, Kampar 31900, Malaysia; e Faculty of Health and Life Sciences, INTI International University, Nilai, 71800 Malaysia; f Faculty of Bioeconomics and Health Sciences, Geomatika University Malaysia, Kuala Lumpur 54200, Malaysia; g Department of Chemistry, College of Science, King Saud University, P.O. Box 2455, Riyadh 11451, Saudi Arabia; h Department of Chemistry and Biochemistry, The Ohio State University, 151 W. Woodruff Ave, Columbus, OH 43210, USA

**Keywords:** Zinc, Phosphorus, Solubilization, Pseudomonas aeruginosa, agricultural productivity, A. hypogaea

## Abstract

Incessant utilization of chemical fertilizers leads to the accumulation of minerals in the soil, rendering them unavailable to plants. Unaware of the mineral reserves present in the soil, farming communities employ chemical fertilizers once during each cultivation, a practice that causes elevated levels of insoluble minerals within the soil. The use of biofertilizers on the other hand, reduces the impact of chemical fertilizers through the action of microorganisms in the product, which dissolves minerals and makes them readily available for plant uptake, helping to create a sustainable environment for continuous agricultural production. In the current investigation, a field trial employing *Arachis hypogaea* L was conducted to evaluate the ability of *Pseudomonas aeruginosa* to enhance plant growth and development by solubilizing minerals present in the soil (such as zinc and phosphorus). A Randomized Complete Block Design (RCBD) included five different treatments as T1: Un inoculated Control; T2: Seeds treated with a liquid formulation of *P. aeruginosa*; T3: Seeds treated with a liquid formulation of *P. aeruginosa* and the soil amended with organic manure (farmyard); T4: Soil amended with organic manure (farmyard) alone; T5: Seeds treated with lignite (solid) based formulation of *P. aeruginosa* were used for the study. Efficacy was determined based on the plant's morphological characters and mineral contents (Zn and P) of plants and soil. Survival of *P. aeruginosa* in the field was validated using Antibiotic Intrinsic patterns (AIP). The results indicated that the combination treatment of *P. aeruginosa* liquid formulation and organic fertilizer (farmyard) (T3) produced the highest biometric parameters and mineral (Zn and P) content of the groundnut plants and the soil. This outcome is likely attributed to the mineral solubilizing capability of *P. aeruginosa.* Furthermore, the presence of farmyard manure increased the metabolic activity of *P. aeruginosa* by inducing its heterotrophic activity, leading to higher mineral content in T3 soil compared to other soil treatments. The AIP data confirmed the presence of the applied liquid inoculant by exhibiting a similar intrinsic pattern between the *in vitro* isolate and the isolate obtained from the fields. In summary, the Zn and P solubilization ability of *P. aeruginosa* facilitates the conversion of soil-unavailable mineral form into a form accessible to plants. It further proposes the utilization of the liquid formulation of *P. aeruginosa* as a viable solution to mitigate the challenges linked to solid-based biofertilizers and the reliance on mineral-based chemical fertilizers.

## 1 Introduction

Organic farming is an indigenous practice followed in the era of agricultural cooperatives. The advent of modern farming techniques like chemical fertilizers, pesticides, and genetic modification techniques led to the decline of organic farming. Chemical pesticides and fertilizers are a crucial part of contemporary agriculture's strategy to improve soil fertility and crop productivity. The use of such chemicals not only boosts crop productivity but also changes the physicochemical and biological properties of the soil, causing a decrease in the amount of soil organic matter (SOM), hardening the soil, reducing important nutrients and minerals, weakening microbial activity in the cropping system, and becoming responsible for the emission of greenhouse gases due to the deposition of applied chemicals. Additionally, owing to these modifications in the ecology of the soil, fertilizer that has been applied often reverts to insoluble forms that are not bioavailable to plants [1]. Although intensive farming techniques are necessary for catering to a growing population, they have adverse effects on consumers as a consequence of the high concentrations of chemical fertilizers, pesticides, heavy metals, nitrates, growth stimulators, and transgenic organisms [2] that cause hemoglobin disorders, stomach and gastrointestinal pains, dizziness, bloody diarrhea, tremors, migraines, mental impairments, redness or itching of the skin and eyes, nausea, vomiting, flushing of the face and cancer [3]. Nowadays people are showing faith in organic farming and are interested in consuming organic products because of their safety and nutrition.

**Table 1:** Effect of different treatments on the root length and shoot length of the groundnut plants.

Treatments	Root length (cm)				Shoot length (cm)			
	30th	60th	90th	120th	30th	60th	90th	120th
**T1**	5.43 ± 0.37^e^	9.58 ± 0.17^e^	12.4 ± 0.43^d^	15.18 ± 0.36^d^	12.525 ± 0.30^d^	22.45 ± 0.52^e^	29.075 ± 0.25^e^	38.175 ± 0.40^e^
**T2**	9.05 ± 0.40^c^	13.1 ± 0.39^c^	15.03 ± 0.26^c^	19.98 ± 0.46^b^	22.475 ± 0.34^a^	24.5 ± 0.35^c^	34.075 ± 0.33^c^	46.45 ± 0.5^c^
**T3**	**12.4 ± 0.34**^**a**^	**17.53 ± 0.33**^**a**^	**19.48 ± 0.18**^**a**^	**30.48 ± 0.45**^**a**^	**22.975 ± 0.17**^**a**^	**32.125 ± 0.42**^**a**^	**46.05 ± 0.42**^**a**^	**57.2 ± 0.49**^**a**^
**T4**	10.45 ± 0.28^b^	16.1 ± 0.25^b^	16.45 ± 0.38^b^	19.1 ± 0.52^c^	19.025 ± 0.41^b^	28.05 ± 0.19^b^	41.5 ± 0.45^b^	48.925 ± 0.51^b^
**T5**	7.03 ± 0.12^d^	12.13 ± 0.41^d^	12.65 ± 0.20^d^	15.5 ± 0.29^d^	17.425 ± 0.37^c^	23.45 ± 0.38^d^	32.1 ± 0.18^d^	42.925 ± 0.63^d^
Treatments: 1725.292*** Days: 3401.657*** Treatments × Days: 138.465 ***					Treatments: 2802.278*** Days: 18232.392*** Treatments × Days: 160.935***			

Values are mean ± SD of four replication samples in each group; Column means followed by common superscript are not significant at 5 % level by DMRT; *** indicates *P* < 0.001; ** indicates *P* < 0.01& * indicates *P* < 0.05 versus control.

T1: Uninoculated Control; T2: Seeds treated with liquid formulation; T3: Seeds treated with liquid formulation and the soil amended with organic manure (Farmyard); T4: Soil amended with organic manure (Farmyard) alone; T5: Seeds treated with lignite (solid) based bioinoculant.

**Table 2:** Effect of different treatments on the fresh weight and dry weight of the groundnut plants.

Treatments	Fresh weight (g)				Dry weight (g)			
	30th	60th	90th	120th	30th	60th	90th	120th
**T1**	7.03 ± 0.67^d^	42.55 ± 1.24^e^	121.68 ± 2.98^e^	133.43 ± 2.35^e^	1.62 ± 0.11^c^	8.66 ± 0.48^e^	42.04 ± 2.19^c^	42.96 ± 1.51^e^
**T2**	18.29 ± 1.28^c^	61.17 ± 1.71^c^	183.59 ± 3.35^c^	301.08 ± 4.36^c^	1.95 ± 0.22^c^	15.77 ± 1.41^c^	57.07 ± 2.10^b^	71.48 ± 1.48^c^
**T3**	**30.82 ± 1.64**^**a**^	**101.92 ± 1.55**^**a**^	**201.67 ± 4.14**^**a**^	**420.57 ± 6.52**^**a**^	**7.58 ± 1.38**^**a**^	**22.71 ± 1.91**^**a**^	**63.17 ± 2.18**^**a**^	**150.22 ± 2.68**^**a**^
**T4**	24.42 ± 1.66^b^	84.08 ± 1.76^b^	193.07 ± 6.11^b^	390.97 ± 7.49^b^	6.23 ± 0.99^b^	18.93 ± 1.44^b^	55.79 ± 2.05^b^	131.85 ± 2.54^b^
**T5**	8.49 ± 0.14^d^	44.90 ± 1.07^d^	141.59 ± 2.27^d^	222.66 ± 5.17^d^	2.12 ± 0.22^c^	11.23 ± 0.86^d^	43.46 ± 1.82^c^	61.44 ± 1.99^d^
Treatments: 2817.257*** Days: 23627.855*** Treatments × Days: 859.861***					Treatments: 1430.534*** Days: 11345.352*** Treatments × Days: 633.787 ***			

Values are mean ± SD of four replication samples in each group; Column means followed by common superscript are not significant at 5 % level by DMRT; *** indicates *P* < 0.001; ** indicates *P* < 0.01& * indicates *P* < 0.05 versus control.

T1: Uninoculated Control; T2: Seeds treated with liquid formulation; T3: Seeds treated with liquid formulation and the soil amended with organic manure (Farmyard); T4: Soil amended with organic manure (Farmyard) alone; T5: Seeds treated with lignite (solid) based bioinoculant.

**Table 3:** Effect of different treatments on the number of pods and pod weight of groundnut plants.

Treatments	No of pods/plant			Pod weight/plant (g)		
	60th	90th	120th	60th	90th	120th
**T1**	31.0 ± 1.63^c^	77.25 ± 2.06^d^	108.5 ± 3.31^e^	13.59 ± 1.36^e^	86.14 ± 3.39^e^	146.87 ± 3.77^d^
**T2**	38.25 ± 1.70^b^	120.25 ± 3.77^b^	156.25 ± 4.11^c^	33.41 ± 2.31^c^	136.24 ± 2.56^c^	189.34 ± 3.08^c^
**T3**	**55.25 ± 2.5**^**a**^	**132.25 ± 2.62**^**a**^	**183.25 ± 2.36**^**a**^	**76.732 ± 3.30**^**a**^	**188.44 ± 1.91**^**a**^	**316.75 ± 3.31**^**a**^
**T4**	52.5 ± 2.64^a^	122.00 ± 2.44^b^	162.25 ± 4.50^b^	57.21 ± 2.80^b^	165.68 ± 3.61^b^	249.61 ± 6.52^b^
**T5**	38.0 ± 2.16^b^	93.25 ± 3.09^c^	140.5 ± 1.91^d^	27.02 ± 2.61^d^	122.47 ± 2.46^d^	188.82 ± 5.30^c^
Treatments: 585.613*** Days: 7189.519*** Treatments × Days: 53.610***				Treatments: 1879.525*** Days: 13117.951*** Treatments × Days: 158.844***		

Values are mean ± SD of four replication samples in each group; Column means followed by common superscript are not significant at 5 % level by DMRT; *** indicates *P* < 0.001; ** indicates *P* < 0.01& * indicates *P* < 0.05 versus control.

T1: Uninoculated Control; T2: Seeds treated with liquid formulation; T3: Seeds treated with liquid formulation and the soil amended with organic manure (Farmyard); T4: Soil amended with organic manure (Farmyard) alone; T5: Seeds treated with lignite (solid) based bioinoculant.

**Table 4:** Effect of different treatments on the yield components of the groundnut plants.

Treatments	100 pod weight per plant (g)	100 seed weight per plant (g)	No.of pods per plant	No.of seeds per plant
**T1**	154.67 ± 3.44^d^	42.77 ± 2.40^c^	108.5 ± 3.31^e^	199.25 ± 1.70^d^
**T2**	168.35 ± 5.05^c^	47.15 ± 4.28^abc^	156.25 ± 4.11^c^	270.25 ± 4.11^b^
**T3**	220.15 ± 6.40^a^	52.07 ± 3.48^a^	183.25 ± 2.36^a^	300.25 ± 4.64^a^
**T4**	176.69 ± 4.89^b^	51.15 ± 4.11^ab^	162.25 ± 4.50^b^	296.25 ± 3.30^a^
**T5**	158.14 ± 4.98^d^	45.97 ± 4.03^bc^	140.5 ± 1.91^d^	259.5 ± 3.41^c^

Values are mean ± SD of four replication samples in each group; Column means followed by common superscript are not significant at 5 % level by DMRT.

T1: Uninoculated Control; T2: Seeds treated with liquid formulation; T3: Seeds treated with liquid formulation and the soil amended with organic manure (Farmyard); T4: Soil amended with organic manure (Farmyard) alone; T5: Seeds treated with lignite (solid) based bioinoculant.

**Table 5:** Effect of different treatments on the zinc and phosphorus content of the seeds of groundnut plants.

Treatments	Zinc (mg/kg)	Phosphorus (mg/kg)
**T1**	1.05 ± 0.28^d^	14.03 ± 0.38^e^
**T2**	3.03 ± 0.32^c^	19.05 ± 0.61^c^
**T3**	**6.04 ± 0.30**^**a**^	**25.08 ± 0.81**^**a**^
**T4**	5.01 ± 0.35^b^	23.28 ± 0.91^b^
**T5**	1.08 ± 0.53^d^	16.17 ± 0.80^d^

Values are mean ± SD of four replication samples in each group; Column means followed by common superscript are not significant at 5 % level by DMRT.

T1: Uninoculated Control; T2: Seeds treated with liquid formulation; T3: Seeds treated with liquid formulation and the soil amended with organic manure (Farmyard); T4: Soil amended with organic manure (Farmyard) alone; T5: Seeds treated with lignite (solid) based bioinoculant.

**Table 6:** Effect of different treatments on the zinc and phosphorus content of the field soil.

Treatments	Mineral content (zinc and phosphorus) in soil							
	Zinc (mg/kg)				Phosphorus (kg/ha)			
	30th	60th	90th	120th	30th	60th	90th	120th
**T1**	1.64 ± 0.09^b^	1.86 ± 0.72^d^	2.04 ± 0.50^c^	2.09 ± 0.78^c^	21.1 ± 1.13^c^	24.90 ± 1.11^d^	27.50 ± 1.60^d^	29.60 ± 0.81^d^
**T2**	1.96 ± 0.16^b^	3.03 ± 0.45^bc^	4.36 ± 0.70^b^	5.38 ± 0.97^b^	27.3 ± 1.75^b^	29.38 ± 1.38^c^	33.10 ± 1.63^c^	37.33 ± 1.58^b^
**T3**	**2.55 ± 0.40**^**a**^	**4.24 ± 0.65**^**a**^	**5.67 ± 0.86**^**a**^	**7.24 ± 0.83**^**a**^	**30.45 ± 1.34**^**a**^	**38.13 ± 1.40**^**a**^	**43.23 ± 1.35**^**a**^	**49.65 ± 1.71**^**a**^
**T4**	2.04 ± 0.14^b^	3.43 ± 0.81^ab^	5.11 ± 0.88^ab^	6.79 ± 1.26^ab^	29.4 ± 1.80^ab^	35.50 ± 1.46^b^	40.33 ± 1.22^b^	47.83 ± 1.22^a^
**T5**	1.79 ± 0.54^b^	2.23 ± 0.33^cd^	2.82 ± 0.60^c^	3.22 ± 0.99^c^	23.18 ± 1.08^c^	26.20 ± 1.41^d^	29.50 ± 1.39^d^	33.15 ± 1.49^c^
Treatments: 50.445*** Days: 65.588*** Treatments × Days: 5.587***					Treatments: 318.205*** Days: 314.457*** Treatments × Days: 9.572***			

*Values are mean ± SD of four replication samples in each group; Column means followed by common superscript are not significant at 5 % level by DMRT; *** indicates *P* < 0.001; ** indicates *P* < 0.01& * indicates *P* < 0.05 versus control.

T1: Uninoculated Control; T2: Seeds treated with liquid formulation; T3: Seeds treated with liquid formulation and the soil amended with organic manure (Farmyard); T4: Soil amended with organic manure (Farmyard) alone; T5: Seeds treated with lignite (solid) based bioinoculant.

**Fig. 1:**
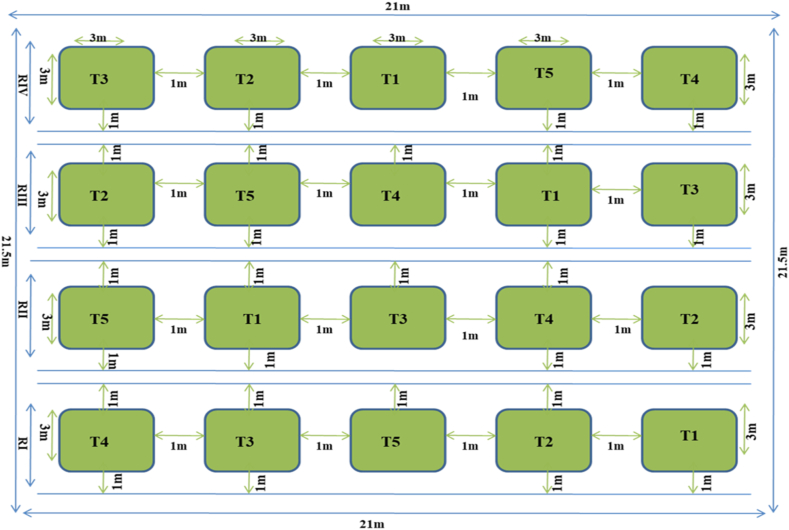
Map showing the Randomized complete block design (RCBD) of field experiments. T1: Uninoculated Control; T2: Seeds treated with liquid formulation; T3: Seeds treated with liquid formulation and the soil amended with organic manure (Farmyard); T4: Soil amended with organic manure (Farmyard) alone; T5: Seeds treated with lignite (solid) based bioinoculant.

**Fig. 2:**
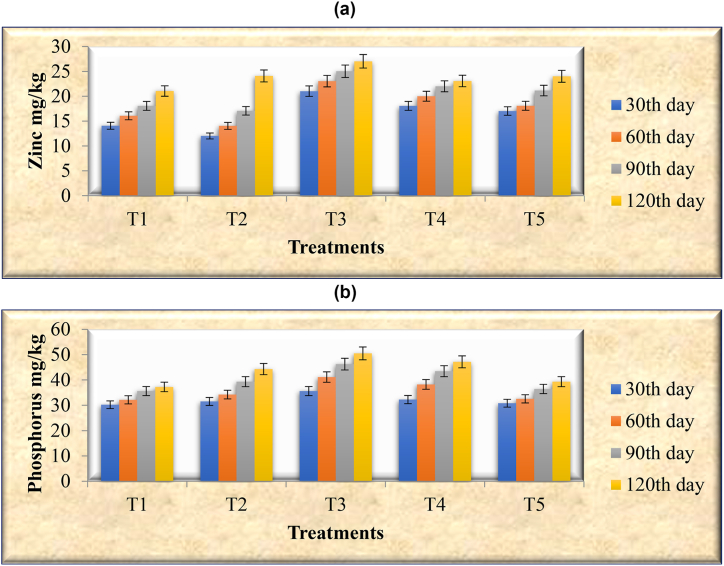
Effect of different treatments on the zinc content of groundnut plants. Effect of different treatments on the phosphorus content of the groundnut plants T1: Uninoculated Control; T2: Seeds treated with liquid formulation; T3: Seeds treated with liquid formulation and the soil amended with organic manure (Farmyard); T4: Soil amended with organic manure (Farmyard) alone; T5: Seeds treated with lignite (solid) based bioinoculant.

**Fig. 3:**
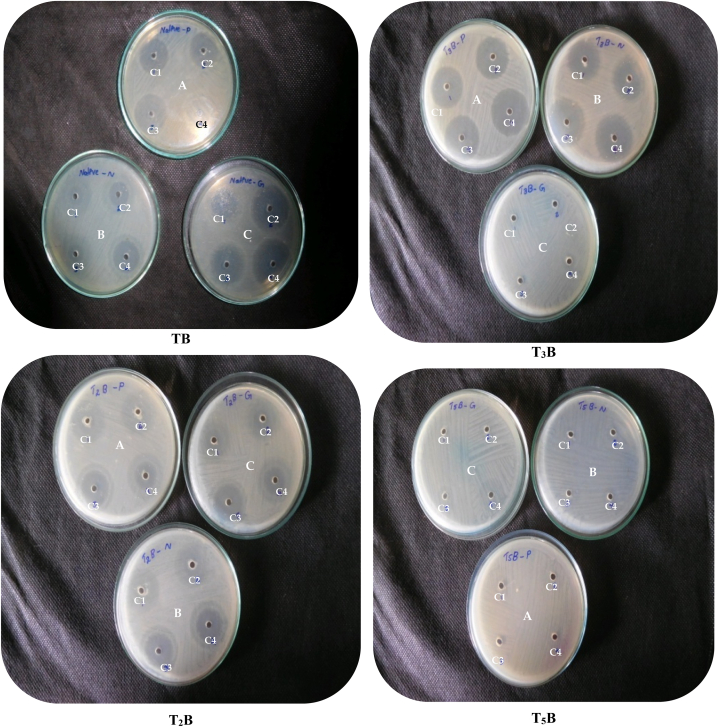
Inhibitory zone obtained by *P.aeruginosa* subjected to three different antibiotics. A: Penicillin; B: Neomycin; C: Gentamycin. C1: 1 μg/ml concentration of antibiotics; C2: 2 μg/ml concentration of antibiotics; C3: 3 μg/ml concentration of antibiotics; C4: 4 μg/ml concentration of antibiotics; TB: *P*.*aeruginosa* isolated from the agricultural field and maintained as pure culture *in vitro*; T_2_B: Bacteria isolated from the soil of T2 plot (Seeds treated with Liquid inoculant (*P*. *aeruginosa*) alone); T_3_B: Bacteria isolated from the soil of T3 plot (Seeds treated with Liquid inoculants (*P*. *aeruginosa*) and the soil amended with organic manure); T_5_B: Bacteria isolated from the soil of T5 plot (Seeds treated with Carrier based inoculant (*P*.*aeruginosa*).

The importance of organic food production practices is to improve biological cycles in the agricultural system, increase soil fertility, diminish various forms of pollution, prevent the practice of chemical fertilizers and pesticides, preserve the intrinsic diversity of food, reduce the socio-environmental impact of food production and to increase the supply of adequate quality food [4]. This agroecological sustainability can be met by encouraging farmers to use biofertilizers, which will improve crop yields and sustainably restore degraded soil structure and fertility. Biofertilizers are a source of plant growth promoting rhizobacteria, which are involved in the conversion of minerals deposited in the soil to make it available to plants through its solubilization mechanisms like organic acid synthesis and sugar production [5]. It is expected that the biofertilizer industry, which had a market size of USD 1.57 billion in 2018, will develop at a compound yearly growth rate of 12.1 % between 2022 and 2027 [6].

Plants require all the essential macro and micronutrients for their survival and productivity. Compared to macronutrients, farmers pay less attention to micronutrients. Among the micronutrients, zinc is a crucial component of more than 300 enzymes [7,8] that plays a vital role in plant life processes such as the metabolism of carbohydrates, proteins, and growth regulators, chlorophyll synthesis, photosynthesis, microspore formation, tolerance against biotic and abiotic stress, oxidative damage and maintenance of the integrity of the biological membranes [[8], [9], [10], [11], [12], [13], [14]]. Zinc deficiency negatively affects crop yield and productivity, including stunted growth, delayed maturity, and poor flower and fruit development due to reduced nutrient utilization efficiency [15]. About 30 % of agricultural soils worldwide are deficient in zinc, causing deficiencies in crops grown in those soils, which in turn leads to zinc deficiency in consumers who consume those crops [9]. An assessment of 2,56,000 soil samples across India showed a 50 % zinc deficiency [16]. It is also expected to increase from 42 % in 1970 to 63 % in 2025 [17].

Phosphorus (P) is the second essential macronutrient required by plants for various metabolic processes such as cell division and growth, energy transport, signaling, etc [18,19]. As it is the primary source of reproductive parts of plants, it should be present in sufficient quantity in the early stage of plant growth [20]. It plays a key role in seed formation, maintaining the quality of fruits, vegetables, and cereal crops, tolerance against winter, and antimicrobial resistance [21,22]. P deficiency leads to stunted plant growth, drooping of petioles and leaflets, shrunken leaves, and leaflets not expanding normally [23].

Agricultural communities use zinc and phosphorus as chemical fertilizers which are converted into plant-unavailable forms like Zn(OH), Zn(OH_2_), ZnCO_3_, and Zn(PO_3_)_4_ due to factors like alkaline soil pH and high phosphorus content of the soil [24,25] whereas P fertilizers converted to water-soluble P as orthophosphate ions H_2_PO^4−^ and HPO^2−^ in soil within a few hours after application [26]. In acidic soils, sorption/desorption processes because these negatively charged P ions firmly cling to the surfaces of minerals that contain positively charged ions, like iron (Fe^3+^) and aluminium (Al^3+^). For the negatively charged P, Fe^3+^ and Al^3+^ serve as the sorption sites (Sato and Comerford, 2005). Additionally, in calcareous soils, these P anions precipitate with calcium (Ca^2+^) resulting in highly insoluble compounds in calcium carbonate crystals. Both processes produce fixed or bound P, which is unavailable to plants and accumulates in the soil [[26], [27], [28], [29]].

Deficiencies in either zinc or phosphorus, or both, might lower crop output since they are antagonistic to one another [30]. A nutrient's availability at modest levels frequently results in deficiencies. In this phenomenon, the antagonistic nutrient (P) is available in such substantial quantities that it induces the other (Zn) to become deficient despite the availability of the antagonist nutrient's marginal to normal levels. When phosphorus and zinc are deficient, it can reduce agricultural yields since the two elements have an antagonistic relationship. Because of the relationship between Zn and P, increased soil phosphate concentrations also result in plant deficiencies in Zn. The H^+^ ions produced by phosphate salts prevent Zn from being absorbed from the solution, which increases Zn adsorption into soil components and renders it unavailable to plants. Due to the fact that farmers typically apply much more P fertilizer than Zn fertilizer, Zn-induced P shortage is a very rare issue. The application of phosphatic fertilizers at high rates to soils with poor or marginal Zn availability is the cause of the P-induced Zn deficit. Four theories have been proposed to provide an explanation for this phenomenon (Wijebandara, 2007) such as P may obstruct the movement of Zn from the roots to the top, Zn concentration may decrease due to the dilution induced by the growth response of P, high P availability might exacerbate Zn deficiency in plant tissues and the Plant cells may have metabolic issues if Zn and P levels are off.

According to Soltangheisi et al. [31], the P/Zn ratio may be a more accurate measure of Zn nutritional status than Zn concentration by itself. Sometimes, Zn-deficient symptoms can worsen due to high P levels in the soil. Insoluble Zn_3_(PO_4_)_2_ may be produced in the soil as a result of co-precipitation of zinc and phosphorus, which lowers the availability of zinc by lowering the concentration of zinc in the soil solution. Zinc deficiency or low zinc concentrations can cause phosphorus uptake and transport to increase in the shoot and leaves, which can make the plant poisonous. When compared to phosphorus, this increase in permeability of the plasma membrane in the root only happened with zinc deficiency and was not seen with other micronutrient deficits [[32], [33], [34], [35]].

According to theoretical calculations, the phosphorus (P) and zinc (Zn) that have accumulated in agricultural soils as a result of fixation are sufficient to support the highest crop yields globally for around 100 years [36]. We must refrain from applying chemical fertilizer frequently, which would be an unfavorable activity for the environment and to maintain the soil's fertility condition [37]. If insoluble metals (Zn and P) in the soil are solubilized and made available to crops by sustainable agricultural practices, a large decrease in the use of zinc and phosphate fertilizers may be possible [[38], [39], [40]]. The application of biofertilizers is a potential approach to improve the soil's microbial state, which influences nutrient accessibility and, ultimately, plant growth [41].

Numerous rhizosphere bacteria have the ability to transform these unavailable forms of metal into available forms through solubilization [42] by the secretion of organic acids [43]. Application of more than one microbe for each metal solubilization is often difficult, because antagonistic activity among the strains may reduce the efficacy of the formulant. Many investigations have demonstrated that in order to promote plant growth and development, the microorganisms that are used as biofertilizers require a variety of plant growth promotion (PGP) traits such as indole acetic acid, phosphate solubilization, siderophore, nitrate, and HCN [44]. Similarly, the current study focuses on the development of such rhizobacteria capable of producing IAA and solubilizing both zinc and phosphorus as biofertilizers would be a potential solution to reduce the above deficiencies in crops and the use of those minerals as chemical fertilizers. The practical application of biofertilizers has not shown effective results in fields as compared to laboratories due to unexpected biotic and abiotic stress. Therefore, biofertilizers that can survive and function in different environments need to be developed.

Biofertilizers provide a biological remediation system, which can mobilize nutrients from an unusable state to a useful form and make them available to plants [45]. According to Afzal and Bano [46], using such fertilizer minimizes the need for expensive chemical fertilizers and creates an eco-friendly method by solubilizing the inaccessible mineral nutrients in the soil and making them available to plants. A suitable carrier ought to provide ideal conditions for the inoculant cells' survival and effectiveness resulting in sufficient shelf life as well as enhanced viability and activity in soil. In most instances, inoculants are available in retail stores in the form of solid carriers [47,48].

The primary limitations of solid carrier-based inoculants arise from the high variability in carrier quality, which is source-dependent, and the undefined and complicated composition of carriers. This has a significant impact on the final product and causes issues with the dosage of the inoculant and storage conditions [49]. Bacteria in carrier-based inoculants are less tolerant of physical stress during storage, notably changes in temperature. The shelf life of the inoculants may be prolonged as they are often vulnerable to contamination [[49], [50], [51]]. Adhesives can be added to inoculant to promote adherence when it is applied to seeds or slurry, but doing so adds time and labor to the process [52]. New inoculant formulations need to be developed to tackle challenges with solid carrier-based formulations, that involve greater durability, no contamination, and convenience of delivery. According to Vora et al. [53], liquid bioinoculants have distinctive formulations that contain not only requisite microorganisms and their nutrients but also specific cell protectants or compounds that promote longer shelf life and resistance to unfavorable conditions. High cell count, no contamination, longer shelf life, improved protection against environmental stress, and increased field efficacy are the benefits of liquid formulation [[54], [55], [56], [57]]. Microorganisms are present in liquid formulations in the form of dormant cysts, and after being applied in the field, the dormant form transforms into active cells. As a result, liquid formulations can now be stored for longer than a year [55,58].

Groundnut (*A. hypogaea* L.) is one of the most significant oilseed and food crops in the world. With an average productivity of 1010 kg ha^−1^, groundnut is the main oilseed crop grown on roughly 6.26 million ha in India. Groundnut production in India is only 1640 kg per acre, which is far below the global average and is primarily caused by a zinc and phosphorus deficit compared to other mineral deficiencies. Their improved development, higher production, and high-quality seeds are dependent on both of these (Zn and P) nutrients [[59], [60], [61], [62]].

Some strains of *Pseudomonas* promote plant growth by making plant available phosphorus, potassium, and zinc from the soil, phytohormone synthesis, HCN, lytic enzymes, and siderophores production. Thus, it might be concluded that the bacterial strains of *Pseudomonas* sp. with their multifunctional properties will attract more attention in the field of biofertilization [63].

In the present investigation, the *in vitro* mineral (Zn and P) solubilization capacity of *P. aeruginosa* (KT148590) was previously studied and reported by the corresponding author [64,65]. Five different treatments were employed in field experiments on *A. hypogaea* L. to investigate the influence of *P. aeruginosa* in two different formulations—both solid and liquid-based—on growth, yield, and the solubilization of minerals (Zn and P) in the soil and make them available to plants.

## 2 Materials and methods

### 2.1 Seeds

TNAU CO-6 variety groundnut seeds were procured from the Department of Oilseeds, Tamil Nadu Agricultural University, Coimbatore, which is a mass selection from the Spanish variety (CS9/ICGS5 cross derivation) and can be harvested in 125–130 days. It is generally cultivated during the rainfed (May–June) season. Seeds were randomly selected and surface sterilized using 0.1 % mercuric chloride before experiments.

### 2.2 Seed treatment with liquid bioinoculant

The liquid formulation of *P. aeruginosa* was prepared by adding 3 % Poly Vinyl Pyrrolidone (PVP) to the Bunt and Rovira broth and maintained at ambient temperature. The surface sterilized seeds were mixed with 5 ml liquid bioinoculant (10^9^ cfu/ml per 100 seeds) and 2 ml of 1 % rice gruel as an adhesive and incubated at ambient temperature in sterile plastic bags. It was then dried overnight under shade.

### 2.3 Seed treatment with solid-carrier (lignite) based bioinoculant

Lignite was obtained from ACC Cement, Madukkarai, Coimbatore, Tamil Nadu, India, and was ground to a fine powder and its pH neutralized using CaCO_3_ and packed in high molecular and high-density polyethylene bags (200 g) and sterilized at 250 °F for 30 min. The culture of *P. aeruginosa* was injected aseptically at the rate of 100 ml (10^9^ cfu/ml) per 200 g of lignite pack and covered with a label at the injecting point [66]. Inoculated packets were thoroughly mixed to ensure uniform absorption of the bacterial cells into the carrier material and incubated at 30 °C for a period of three days. Thus, the disinfected seeds were treated with 5 g of solid-based inoculant (10^9^ cfu/ml per 100 seeds) and 2 ml of 1 % rice gruel as an adhesive and incubated at ambient temperature in sterile plastic bags.

### 2.4 Field studies to evaluate the efficiency of *P. aeruginosa* in groundnut crops

The influence of the liquid formulation of mineral solubilizing bacteria on the growth and yield of *A. hypogaea* L was assessed by conducting a field trial under irrigated conditions in an agricultural farm at Kangeyam, Tirupur District, and Tamil Nadu. The experimental plot was laid out in a randomized complete block design (RCBD) with five treatments (T1: Uninoculated Control; T2: Seeds treated with liquid formulation; T3: Seeds treated with liquid formulation and the soil amended with organic manure (farmyard); T4: Soil amended with organic manure (farmyard) alone; T5: Seeds treated with lignite (solid) based bioinoculant) replicated four times with a plot size of 3 m × 3 m (Figure: 1). The spacing between seeds in a row and the inter-row spacing (between rows) were 30 cm. Before planting, the field was leveled and a total of 20 plots were made. One meter space between the replicates was maintained so that any likely interaction effect would be inhibited. Water streams and drainage were created for each plot. The plots were irrigated at 15 days intervals during the growing season. Groundnuts were harvested 120 days after planting.

#### 2.4.1 Biometric characterization of the groundnut plants

The following parameters were observed.

##### 2.4.1.1 1a. Root length and shoot length

Root length [67] and shoot lengths of the randomly selected plants were measured at regular intervals of 30 days after germination and expressed in centimeters (cm).

##### 2.4.1.2 1b. Fresh weight and dry weight of the plants

Plant samples were collected at regular intervals of 30 days after germination. The fresh weight of the plants was measured by means of a digital balance and dried at 70 °C for 6 h to measure the dry weight of the plants and expressed in gram (g).

##### 2.4.1.3 1c. Yield components

The number of pods and seeds per plant was counted and expressed as numbers. 100 pods and 100 seeds were weighed using digital balance and expressed in g.

#### 2.4.2 Estimation of zinc and phosphorus content of the treated soil

##### 2.4.2.1 2a. Estimation of available Zn in soil [68]

10 g of soil was taken in 100 ml of Erlenmeyer flask and 20 ml of 0.005 M solution of diethylene triamine penta acetic acid (DTPA) was added (soil: DTPA 1: 2 ratio) and shaken in a rotary shaker for 2 h. The contents were filtered through Whatman No: 42 filter paper and extracts were collected. This extract was directly fed into the Atomic Absorption Spectrometer (AAS) for determining the concentration of available Zn in the soil.

Preparation of DTPA extraction solution.

The DTPA extraction solution was prepared by dissolving 149.2 g of 0.1 M triethanolamine, 19.67 g of 0.005 M diethylene triamine penta acetic acid, and 14.7 g of 1 M CaCl_2_.2H_2_O in 200 ml of distilled water approximately and then made up to 10 L. The pH was adjusted to 7.3 ± 0.05 using 1 N HCl.

##### 2.4.2.2 2b. Estimation of available phosphorus (P) in soil

The available P in soil was estimated by Olsen's method [69].

#### 2.4.3 Reagents preparation

i. Sodium bicarbonate (0.5 M)
ii. Activated carbon

## 3 iii. 5 N Sulphuric acid

### 3.1 Conc.H_2_SO_4_, 137 ml was added in 1 L of distilled water

#### 3.1.1 Reagent A

a) Ammonium molybdate, 12 g was dissolved in 250 ml of distilled water
b) Antimony potassium tartrate, 0.291 g was dissolved in 100 ml of distilled water
c) 100 ml of 5 N H_2_SO_4_ was prepared by dissolving 137 ml of conc.H_2_SO_4_ in 1 L of distilled water
d) The 3 reagents were mixed as prepared above and the volume was made up to 2 L with distilled water.

## 4 Reagent B

### 4.1 Ascorbic acid, 1.056 g was dissolved in 200 ml of reagent A

#### 4.1.1 Procedure

5 g of soil was taken in 100 ml of Erlenmeyer flask and one teaspoon of activated carbon was added followed by the addition of 50 ml of 0.5 M sodium bicarbonate. The contents were shaken for 30 min in an orbital shaker and filtered through Whatman No: 40 filter paper. More activated carbon was added if necessary to obtain a clear filtrate. 5 ml of filtrate was pipetted into a 25 ml volumetric flask and acidified to pH 5.0 with 5 N H_2_SO_4_. The contents were diluted to 20 ml to which 4 ml of freshly prepared reagent B was added and the volume was made up to 25 ml with distilled water the flask was shaken well and allowed to stand for 10 min. The absorbance of the blue color developed was read in a Vis-Spectrophotometer at 660 nm. A blank was run simultaneously with distilled water. The unknowns were calculated from the standard graph and the available P was expressed in mg/l.

#### 4.1.2 Analysis of zinc and phosphorus content of the groundnut plants

To analyze the zinc and phosphorus content of whole groundnut plants and seeds, they were oven-dried at 70 °C and ground to a fine powder with a Wiley mill. 0.1 g of sample was placed in a 100 ml conical flask with 10 ml of Nitric acid: Perchloric acid in the ratio 9: 4. The whole plant material was placed on a hot plate and digested at 100 °C until it became colorless. The extract was taken in a 50 ml volumetric flask and made up to 50 ml with distilled water. Then the sample was fed to an atomic absorption spectrometer (AAS) (Shimadzu 7000AA) to find the concentration of available zinc present in the sample [63]. The phosphorus content of the samples was estimated following the procedure of Bray and Kurtz [70] as described in section 2.4.2.

#### 4.1.3 Assessment of survival of applied liquid inoculant in the fields using antibiotic intrinsic pattern

To study the survival of applied liquid inoculants in the fields, a comparative study of intrinsic antibiotic sensitivity or resistivity pattern of standard *(in vitro*) isolate *P. aeruginosa* and culture of liquid inoculants obtained from the field after an application was tested by an antibiotic well method. The cultures were made on nutrient agar medium with bacterial suspension inoculations of the medium before plating. After plating and solidification of the medium, antibiotics (neomycin, penicillin, and gentamycin) with four different concentrations such as 1, 2, 3, and 4 μg/ml were poured into the well-impregnated on the solidified agar medium and incubated at 28 ± 1 °C for 24 h and a control plate without antibiotic was also plated. Data for antibiotic sensitivity/resistivity was recorded by measuring the diameter of the growth inhibition zone around the well after 24 h of incubation. The isolates were considered as sensitive (S) or resistant (R) to an antibiotic by comparing it with the data given by the manufacturer. Based on the intrinsic pattern obtained similarities between the organisms can be identified.

### 4.2 Statistical analysis

Statistics were analyzed by ANOVA (Two-way analysis) and compared with Duncan's Multiple Range Test (DMRT) at P ≤ 0.05 using SPSS-19 software.

## 5 Results and discussion

Plants require all essential nutrients in balanced proportion and deviation from this may result in mineral disorders. Of the several micronutrients that increase plant growth and productivity, zinc plays a pivotal role. Both the quantitative and qualitative yield of the plant is strongly dependent on this micronutrient. Supplementation of zinc (Zn) in the form of synthetic fertilizer is proved to be inappropriate due to its unavailability to plants.

Phosphorus (P), the second important macro-nutrient plays a vital role in plant progression and is considered as the most significant growth-limiting factor for many crop productions in India due to its limited availability in the soils. Approximately 70–90 % of P fertilizer applied to the soil gets converted into insoluble forms due to the presence of Fe and Al in acidic soils and Ca in neutral and alkaline soils [71] resulting in poor availability to plants. Accumulation of P due to the regular application of phosphatic fertilizers is also regarded as a factor responsible for zinc deficiency in soil and plants.

Zinc is absorbed by plants as Zn^2+^ and P as H_2_PO_4_^−1^ or HPO_4_^−1^. Positively and negatively charged ions have an electrical attraction to one another, facilitating the formation of a chemical bond either in the soil or the plant tissue. The relative strength of the P–Zn bond is strong and does not readily break without dramatic changes in the physical or chemical environment. If excess P binds a large amount of Zn normally available to the plant, the result can be a P-induced Zn deficiency [72]. This crisis can be averted by using Zn and P solubilizing bioinoculants which have the potential to convert various forms of unavailable metal into available forms to overcome its deficiency in plants, restore soil fertility and achieve organic farming principles. Application of this strain in the form of liquid biofertilizers to crops adds more benefits such as improved shelf life, stable high cell count, high enzymatic efficiency, high ability to combat native population, and resistance to abiotic stresses [72]. Therefore, in the present study, a liquid formulation of *P. aeruginosa* on the growth and yield of *A. hypogaea* L. was evaluated.

### 5.1 Effect of different treatments on the root length and shoot length of the groundnut plants

The root and shoot length of the randomly selected plants subjected to different treatments were measured at regular 30 days intervals after sowing. There was a significant increase in the root and shoot length in all the treatments compared to the control due to the application of bioinoculant. According to the comparative account of overall treatments, combined treatment of organic manure and liquid inoculant (*P. aeruginosa*) of T3 plots showed maximum shoot (57.2 cm) and root length (30.48 cm) on the 120th DAS (day after sowing) followed by other treatments and control (Table: 1). This may be due to the plant growth-promoting activity of bioinoculant (*P. aeruginosa*) that attributed to increase in the photosynthetic activity of crop plants which resulted in the enhancement of vegetative growth. Zinc act as a co-factor for many enzymes [73] and is essential for the synthesis of a growth-promoting substance (Auxin) which stimulates plant growth [74]. Additionally, IAA synthesis has been found in our bacterial isolates, a phytohormone that extends root hairs and could enhance soil nutrient uptake [63]. Phosphorus being an essential constituent of cellular proteins and nucleic acid encourages the meristematic activities in plants. Similarly, Glick et al. [75] reported that *Pseudomonas* strains have increased root and shoot elongation in canola, lettuce, and tomato by their plant growth-promoting activity. Therefore, in the present study *P. aeruginosa* along with the organic manure enhanced plant growth by increasing the availability of essential nutrients (Zn and P) and by synthesizing plant growth-promoting substances such as IAA.

### 5.2 Effect of different treatments on the fresh weight and dry weight of the groundnut plants

A study on the influence of the liquid formulation of mineral solubilizing bacteria (*P. aeruginosa*) on the fresh and dry weight of the groundnut plants showed that plants from T3 plots recorded the highest fresh and dry weight compared to other treatments (Table: 2). This may be due to the metal-solubilizing activity of *P. aeruginosa*, which improves the availability of P, leading to the development of an extensive root system that enables plants to absorb water and nutrients from deep in the soil. This improved the plant's ability to produce more assimilates, which was reflected in higher dry weight [76]. Similar increases in plant parameters were observed in different crops inoculated with *Pseudomonas, Azospirillum, and Azotobacter* strains [77,78]. This finding is in agreement with the reports of Arshad and Frankenbcrgcr, [79]; Biswas et al., [80]; Adesemoye et al.*,* [81] who stated that the application of zinc solubilizing bacteria in the soil can enhance plant growth through metal solubilization and production of plant growth promoters (IAA).

### 5.3 Effect of different treatments on the yield components of the groundnut plants

Zinc (Zn) plays a key role as an activator of several enzymes in plants and is directly involved in the biosynthesis of growth substances such as auxin which produce more plant cells and more dry matter that in turn will be stored in seeds as a sink which leads to increase in yield components [82]. Similarly, phosphorus (P) forms an important nutrient for all crops because it is a key constituent of ATP and plays a significant role in energy transformations in plants and in various forms of seed formation [83]. P increases groundnut yield through the increase in total dry matter [84,85]. Thus, in the present study effect of mineral (Zn and P) solubilizing activity of *P. aeruginosa* on the pod and seed yield of groundnut plants was studied after 60 DAS (days after sowing). Among the different treatments, plants of T3 plots showed maximum pod number (183.25/plant) and pod weight (316.75 g/plant) compared to control on the 120th day after sowing (Table: 3). The other yield components such as the hundred pod weight, hundred seed weight and the number of pods and seeds per plant were also evaluated. In all the parameters studied plants of T3 plots showed maximum value (Table: 4). These results may be attributed to the nature of root exudates which act as suitable substrates for the associative microorganisms that release plant growth-promoting substances mainly indole-acetic acid. These results stand in accordance with those obtained by Kloepper [86], Tilak et al. [87] and Verma et al. [88]. Similarly, the phosphate-solubilizing and phytohormone-producing activity of *Azotobacter chroococcum* showed an increase in the grain and straw yield of wheat [89]. Stimulation in the growth and yield of maize by inoculation with *Rhizobium leguminosarum* and *Penicillium rugulosum* under glasshouse and field conditions were also reported by Chabot et al. [90]and Reyes et al. [91]. Zinc solubilizing activity of Bacillus sp. AZ6 improved the maximum growth and physiological parameters of maize seedlings which might be due to the growth-promoting attributes compared to other isolated strains [92]. Srithaworn et al. [93], also revealed that inoculation with P. megaterium KAH109 and P. aryabhattai KEX505 considerably increased plant dry weight by 26.96 % and 8.79 %, respectively, and the number of grains per plant by 48.97 % and 35.29 % when compared to those of the uninoculated control and concluded that both strains can be considered as a potential zinc solubilizing bioinoculant to promote the growth and production yield of green soybeans.

Liquid inoculants along with organic manure (T3) promoted more growth and yield of groundnut plants compared to liquid inoculant (T2) and organic manure (T4) alone because organic manure helped in the proliferation of microorganisms in soil by providing essential nutrients required for mineralization activity that result in slow release of nutrients to crop leading to enhanced growth and yield of groundnut crops.

Seed treatment with a lignite-based formulation of *P. aeruginosa* (T5) recorded the least growth and yield of groundnut plants compared to the treatment involving liquid inoculant. This might be due to the poor viability and inconsistent field performance of solid carrier-based bioinoculant compared to liquid formulation. The enhanced performance of liquid inoculant in the field is due to the fact that, as concentrations of salts increase in the cell environment with the drying of liquid inoculant, stabilizing polymers such as PVP may be useful in reducing the extent of protein precipitation or coagulation of cells. Maintenance of macromolecular structure may improve biological integrity, thus leading to improved survival and field performance [94]. Tittabutr et al. [56] reported that liquid inoculants formulated with polymeric additives promoted the long-term survival of all rhizobial strains. The result is also related to Girisha et al. [95] who concluded that liquid *Rhizobium* inoculants prepared with PVP as an osmoprotectant had improved shelf life, nodulation, and nitrogen fixation on par with lignite-based inoculants in cowpea.

Thus, the present study clearly demonstrated that inoculation with plant growth-promoting rhizobacteria (*P. aeruginosa*) significantly enhanced the growth and yield of groundnut.

### 5.4 Analysis of zinc and phosphorus content of the groundnut plants and the soil

The effect of mineral solubilizing bacteria on the zinc content of groundnut plants and seeds of different treatments and its effect on the availability of zinc in the soil was studied. Findings showed that the available zinc content was significantly (*P* < 0.05) higher in the soil, plants, and seeds of the T3 plot compared to other treatments (Figure: 2.1 & 2.2; Table: 5–6). This may be due to the solubilization of insoluble Zn in soil by *P. aeruginosa* through the production of gluconic acid. This was anticipated since increasing soil-available zinc could lead to higher zinc levels in the plants Sethia et al.[ [96]. Numerous investigations using PGPRs have also shown that this increase in zinc concentration is beneficial. According to Lefèvre et al. [97], PGPRs have been shown to increase zinc translocation in wheat grains by 12 % above artificial zinc, overcoming nutrient deficiencies in numerous crops. Simine et al. [98] reported that gluconic and 2-keto gluconic acid production by the strain of *Pseudomonas fluorescens* was responsible for Zn solubilization in broth assay. Praveen Kumar et al. [99] further reinforce this fact by examining the zinc content of maize plants grown in the presence of ZnSO_4_ with rhizobacteria and concluded that the plant uptake of readily available soil zinc source (ZnSO_4_) was insufficient, bacterial treatment is therefore required to mobilize soil mineral elements, which leads to an increased zinc lev-el in maize plants through its solubilization mechanisms. Ramesh et al. [100] also reported that Zn solubilizing strains of *Bacillus aryabhattai* enhanced Zn accumulation in wheat and soybean. Abaid-Ullah et al. [101] compared Zn translocation in wheat grains with chemical Zn and found that certain strains of *Serratia* sp., *Pseudomonas* sp., and *Bacillus* sp., enhanced to 7–12 % over the chemical. Sirohi et al. [102] also found that the application of *Pseudomonas fluorescens* strain (PSd) enhanced Zn2+ content in wheat plants and soil by its zinc solubilizing activity. Roesti et al. and Mader et al. [103,104] reported that inoculation of *Pseudomonas synxantha* HHRE81 (R81) and *P. jessenii* LHRE62 (R62) increased zinc concentration in wheat and black gram seeds. In accordance with Vaid et al. (2014) [105], rice plants inoculated with an appropriate combination of *Burkholderia* sp. and *Acinetobacter* sp. Zn-solubilizing bacterial strains were also found to be more effective than uninoculated plants at acquiring Zn from Zn-deficient soil. The application of organic manure in the T3 plot was also considered as a factor for the maximum Zn availability in soil and plants. Organic manure is involved in promoting plant growth, which induces the activity of *P. aeruginosa* by providing essential nutrients. A similar observation was made by several researchers that the application of different organic materials along with biofertilizers increased Zn solubility and uptake by plants [106].

The effect of the mineral solubilization capacity of *P. aeruginosa* on the P content of the groundnut plants, seeds, and soils of different treatments was studied. Among different treatments, soils, plants, and seeds of T3 plots recorded higher accumulation of P compared to other plots (Table: 5–6). This may be attributed to the plant growth-promoting activities of *P. aeruginosa* such as P-solubilization and IAA production. A similar observation was supported by studies from Refs. [[107], [108], [109], [110]] reported that the maximum increase in P uptake and consequent plant yield could be attributed to the ability of 10.13039/100016706PSB strains to solubilize insoluble inorganic phosphates and produce required phytohormones. These results suggest that P solubilizers increase soil P content and enhance P uptake in many crops [[111], [112], [113]]. Pal [114] reported that the phosphate nutrition of maize, finger millet, amaranthus, and buckwheat was improved after seed inoculation of crops with phosphate-solubilizing Bacillus sp. Inoculation of PSB such as Serratia marcescens, Pseudomonas fluorescens, and Bacillus sp. increased P uptake in maize and peanut plants [60,115,116]. Organic manure applied in the T3 plots also supported the increase of P content in the soils and plants due to its heterotrophic activity. This result is in agreement with Aman-ullah et al. [117] suggested that soil organic matter improves soil physical properties and contributes to the formation of soluble complexes with metal ions (natural chelates) which ultimately enhance the uptake of these metal ions by plants.

### 5.5 Assessment of survival of applied inoculant in the fields using antibiotic intrinsic pattern

Determining the dynamics of root colonization by the introduced bacteria is essential for their effective use, as it is critical in plant growth promotion and biological control [118]. Many bacteria are intrinsically resistant or sensitive to various antibiotics. The range and the concentration of antibiotics to which these bacteria are resistant or sensitive, varies considerably, even among strains within the same species. This unique pattern of intrinsic antibiotic resistance or sensitivity can be applied as a genetic fingerprint of an organism and used to recognize it [119]. Thus in the present study, the survival of inoculated liquid inoculant (*P*.*aeruginosa*) in the groundnut field was assessed by comparing the intrinsic antibiotic patterns obtained by the standard *in vitro* strain (*P*.*aeruginosa*) (TB) and the strains (T2B) obtained from the rhizospheric soil samples of groundnut plants harvested on 120 DAS from the plots of T2 [Seeds treated with Liquid formulation (*P*.*aeruginosa*) alone)], T3B [Bacteria isolated from the soil of T3 plot (Seeds treated with Liquid formulation (*P*.*aeruginosa*) and the soil amended with the organic manure)] and T5B [Bacteria isolated from the soil of T3 plot (Seeds treated with solid-carrier (lignite) based inoculant (*P*.*aeruginosa*)]. All the isolates showed resistivity to all intrinsic antibiotics tested (neomycin, penicillin, and gentamycin) at the concentration of 4 μg/ml and showed similar intrinsic patterns which confirmed that all the isolates were similar (Figure: 3). Similar investigation was made by Laxmi prasuna [120] who characterized rhizobium isolates associated with wild legumes on the basis of antibiotic resistance and concluded that isolates showed sensitivity towards ten antibiotics (chloramphenicol, erythromycin, gentamycin, kanamycin, neomycin, nystatin, oxytetracycline, penicillin G, streptomycin and vancomycin) which presented a picture that differentiates each of the isolates from other and appeared a useful criterion to distinguish them at this level while evaluating the biology of ‘*Rhizobia*’. Mazumdar et al. [121] also used nine fluorescent pseudomonas isolates obtained from the rhizosphere of tea plants for the determination of Intrinsic Antibiotic Resistance Profile (IARP) using six antibiotics such as kanamycin, streptomycin, rifampicin, gentamycin, ampicillin, and chloramphenicol. Most of the isolates showed high resistance against two antibiotics, ampicillin, and kanamycin and it was concluded that intrinsic levels of resistance patterns to a particular class of antibiotics can be used for strain identification.

## 6 Conclusion

The current research demonstrated the effectiveness of a liquid formulation of a mineral-solubilizing bacteria (*P. aeruginosa*) along with farmyard manure-enhanced mobilization of minerals (Zn and P) that were accumulated in the soil because of the continuous use of chemical fertilizer through its solubilization mechanism and stimulated plants uptake for their growth and development. The study provides a strategy for restoring productive soil and minimizing the use of such minerals as chemical fertilizers. The use of bio-fertilizers should be made obligatory in agriculture to establish a sustainable agricultural environment, minimize the losses brought on by current farming practices, and provide organic food enthusiasts with wholesome, non-toxic food.

## Data availability statement

• Data included in article/supplementary material/cited in the article.
• Data will be made available on request

## CRediT authorship contribution statement

**K. Sunitha kumari:** Validation, Formal analysis, Data curation, Conceptualization. **S.N. Padma Devi:** Validation, Formal analysis, Data curation, Conceptualization. **Rajamani Ranjithkumar:** Validation, Methodology, Formal analysis, Data curation, Conceptualization. **Sinouvassane Djearamane:** Validation, Methodology, Formal analysis, Data curation, Conceptualization. **Lai-Hock Tey:** Investigation, Funding acquisition, Investigation, Funding acquisition. **Ling Shing Wong:** Visualization, Investigation, Funding acquisition. **Saminathan Kayarohanam:** Supervision, Project administration, Funding acquisition. **Natarajan Arumugam:** Supervision, Project administration, Investigation, Funding acquisition. **Abdulrahman I. Almansour:** Project administration, Funding acquisition. **Karthikeyan Perumal:** Supervision, Project administration.

## Declaration of competing interest

The authors declare the following financial interests/personal relationships which may be considered as potential competing interests.
